# Best Practices in the Conduct of Community Engagement Studios at MaineHealth Institute for Research

**DOI:** 10.46804/2641-2225.1238

**Published:** 2025-12-18

**Authors:** Francesca Piccolo, Emma DayBranch, Carolyn Sullivan, Elizabeth Woods, Neil Korsen, Jan Carney, Jenna Schiffelbein, Kathleen Fairfield

**Affiliations:** a Center for Interdisciplinary Population and Health Research, MaineHealth Institute for Research, Westbrook, Maine; b Larner College of Medicine, University of Vermont, Burlington, Vermont; c Dartmouth Cancer Center, Lebanon, NH

**Keywords:** Translational research, Research design, Referral and consultation, Community-based participatory research, Feasibility studies

## Abstract

**Problem Statement:**

Community engagement (CE) Studios are an established method for integrating lived-experience expertise into research at 1 or more specific points within a project. Although many researchers understand the value of incorporating community input, they often lack the tools, skills, or infrastructure to do so effectively. CE Studios address this challenge by offering a structured, consultative approach to engagement.

**Background:**

CE Studios occur at any point across the research lifecycle and are used to inform research design, feasibility, relevance, recruitment, implementation, and translation. In a 1-time, 2-hour consultation, CE Studios bring lived-experience expertise into the research process, helping projects center patients’ perspectives and inform research direction. Rooted in prior work by the Vanderbilt Institute for Clinical and Translational Research, this model builds rapport with intended audiences and strengthens capacity for engagement among researchers and community members.

**Application:**

CE Studios require institutional support through the establishment of a neutral team trained in the model. At MaineHealth Institute for Research, a CE Studios team comprising community engagement and outreach navigators and staff was established in fall 2022. The CE Studio program is supported by the Northern New England Clinical and Translational Research Network infrastructure grant and nested within a larger community-engaged research effort. Since launch, the team has supported 14 CE Studios for 11 projects and collaborated with 5 institutions across Northern New England. This paper describes the implementation process at MaineHealth Institute for Research, as well as team roles, CE Studios structure, and lessons learned from launching and sustaining the model.

## 1. Problem statement

Incorporating patient and community voices into research may help shorten the gap between research and its translation into practice.^1^ Engaging with community can improve research relevance and feasibility, as well as increase understanding, trust, and traction in disseminating research.^2^ Rural communities face additional challenges in research translation that stem from geographic isolation, values of independence, limited social connectivity, and mistrust toward research—which make meaningful engagement particularly difficult.^3^ Maintaining strong partnerships between academic centers and communities, especially rural ones, can help facilitate engagement, improve research translation, and inform future directions.

Although many researchers recognize the value of including patient and community perspectives in research development, operationalizing this notion can be challenging.^4^ Few researchers are equipped to identify, recruit, convene, or appropriately onboard community members with lived experience as advisers. The Community Engagement (CE) Studio model was developed to address these common engagement barriers.^4^

CE Studios are a method best positioned to support engagement as consultation. CE Studios are not designed for higher amounts of engagement, such as collaboration or shared leadership, because the activity is a 1-time meeting in which decision-making power and resources (beyond stipends) remain with the research team.^5^ However, they can be used within broader community-engaged research (CER) or community-based participatory research frameworks in which other mechanisms support community engagement or partnership with higher involvement.^6^

## 2. Background

CE Studios are designed to bring together the perspectives of community members (referred to as “community experts”) to research projects. This approach allows researchers to engage with interest holders throughout the research process and gain critical feedback from communities of interest at 1 or more specific point(s) across the research lifecycle. CE Studios can be conducted in-person or virtually. For the purposes of this paper and our application in a rural population, we describe CE Studios in a virtual format and highlight the support person in a tech-support role.^7^

### 2.1. Origin

The Meharry-Vanderbilt Community Engaged Research Core (a shared resource developed by the Vanderbilt Institute for Clinical and Translational Research) developed the CE Studios framework to help non-research interest holders provide feedback to the research team before project implementation.^8^ Since their development in 2009, CE Studios have been adopted, adapted, and applied by numerous teams and institutions across the country.^9–12^ This method depends on the development of a neutral CE Studios team and requires institutional support. Though CE Studios have some similarities to both focus groups and research studios, these models are unique and serve different purposes.

### 2.2. CE studios process and roles

#### 2.2.1. Overall process

The CE Studios activity is a 1-time, highly structured meeting that typically lasts 2 hours and is meant to inform a research project by collecting input from community members who represent the research population of interest. Each meeting is supported and facilitated by a CE Studios team that consists of a facilitator, support person, and notetaker. CE Studios are started when the research team submits a request.

Once a CE Studio has been requested, the CE Studios team meets with the research team to assess project priorities and factors, and to determine whether a CE Studio is an appropriate engagement tool. Relevant lived experience is defined by the research team often during the initial meeting. The CE Studios team recruits 8 to 10 community members with relevant lived experience to serve as community experts and orients them to the activity. Collaborations with community-based organizations (CBOs) can help recruit the target audience for the CE Studio. In parallel, the CE Studios team supports the researcher in developing a lay-friendly presentation about their research and a CE Studios facilitation guide designed to solicit input on 2 to 3 areas of the project.

**Fig. 1.**
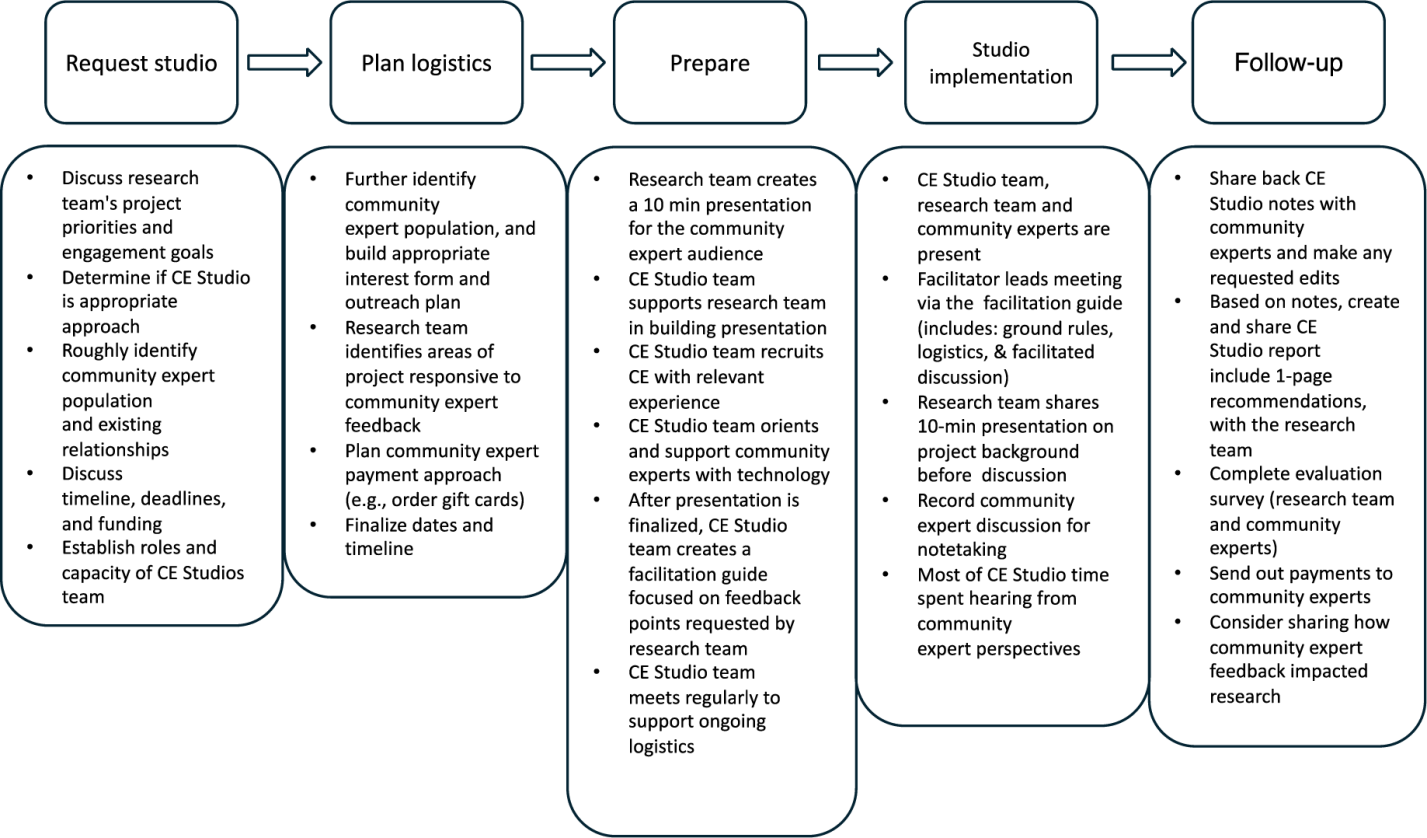
Community engagement studio process at MHIR. Adapted from Vanderbilt University.^8^ CE, community engagement.

The CE Studio is then conducted with distinct roles for all involved (Table 1). Each CE Studio starts with an initial brief orientation, a review of ground rules, and introductions. Then, the research team presents necessary background information. The remaining time is spent in a facilitated discussion of feedback and insights from the community experts. After the meeting, the CE Studios team ensures community experts receive payment, a copy of the notes, and opportunities to provide any additional feedback. The CE Studios team then synthesizes the findings, delivers a recommendations report to the research team, and assists in considerations for further engagement. Evaluation surveys are sent to community experts and research teams. The entire process usually takes 12 to 14 weeks, with most of the time spent on recruitment (Fig. 1).

#### 2.2.2. Primary research team

This team can include the principal investigator, co-principal investigator, and research coordinator or assistant. The CE Studios usually limits the research team to 2 members during the CE Studios meeting to ensure most participants are community experts.

#### 2.2.3. CE Studios team

At MHIR, the CE Studios activity is supported through 3 key roles: a facilitator, a notetaker, and a support person. In the Vanderbilt model, these roles are defined as facilitator, community navigator, and faculty.^8^

#### 2.2.4. Facilitator

The facilitator has a neutral role and guides the CE Studios for the research team and community experts. They hold a welcoming space for the community experts’ perspectives, support inclusive discussion, and maintain structure. Facilitators are trained in facilitation techniques and may also have experience with the population of interest.

#### 2.2.5. Notetaker

The notetaker documents key recommendations, consensus, and feedback. Notes are reviewed by community experts before being used to form a 1-page report of highlights and recommendations. Both recommendations and notes (once reviewed by community experts) are shared with the research team. With community expert permission, we have used recordings to enhance notetaking.

#### 2.2.6. Support person

The support person is responsible for ensuring that all technical issues are resolved.^7^ They ensure that the video-conferencing method runs smoothly, community experts can access the call, and support features (eg, chat, raised hands, muting/unmuting) are actively monitored. One-on-one meetings with tech support and a contact number are also provided in advance in case community experts need assistance.

#### 2.2.7. Community experts

Once the relevant lived experience is identified, flyers linked to an interest form are used to recruit for the activity. Regional CBOs, partners, and networks often help with this recruitment. The interest form helps to ensure community experts have the relevant lived experience, technology, and availability to participate. CE Studio teams reach out to interested and eligible individuals, orient them to the activity, answer any questions, and support them with any technology or logistic needs before the meeting. All lived-experience experts are compensated for their time and expertise.

### 2.3. CE studios vs focus groups and research studios

It is important to distinguish CE Studios from other common research activities, such as focus groups or research studios, to ensure appropriate application and understanding.

#### 2.3.1. CE Studios vs focus groups

The primary difference between a CE Studio and focus group is that a CE Studio is not an avenue for data collection or research with human participants. Rather than collecting qualitative data, the purpose a CE Studio is to support, improve, or enhance research design, implementation, translation, or dissemination.^1,13,14^ CE Studio community experts are not research participants; they are experts in their lived experience who are being consulted with the purpose of improving research and its translation. As such, CE Studios do not require institutional review board approval or informed consent. At MHIR, the institutional review board has a standing “not research” determination for this activity. Mechanisms throughout the activity ensure this distinction is maintained (Table 2). Another key difference between CE Studios and focus groups is that CE Studios are bidirectional discussions, whereas focus groups are meant to gather data and are, therefore, unidirectional.^2,4^

#### 2.3.2. CE studios vs research studios

Although both CE Studios and Research Studios are structured activities designed to strengthen research projects, they differ in purpose and type of expertise. Research Studios typically involve members of the broader research community and methodological experts who provide technical feedback on study design, implementation plans, and analytic approaches. These sessions focus on improving scientific rigor and feasibility. In contrast, CE Studios center on the perspectives of community experts with lived experience relevant to the topic. CE Studios and Research Studios can complement each other by offering distinct types of expertise. At MHIR, both CE Studios and Research Studios are available to investigators through the Northern New England Clinical and Translational Research Network (NNE-CTR) infrastructure grant (Table 2).

## 3. Application

Our CE Studios team was established in fall 2022. The team contracted with an independent consultant to train MHIR community navigators and staff on the CE Studios model and provide ongoing mentorship as the team built proficiency.

The MHIR CE Studios team comprises community engagement and outreach (CEO) navigators and staff. The CE Studios program is supported by the NNE-CTR infrastructure grant and nested within a larger CER effort. This approach ensures CE Studios occur within the context of building and sustaining community-academic partnerships across the rural region. The NNE-CTR team has 3 place-based CEO navigators living and working in 2 rural Maine communities and 1 Vermont community. The CEO navigator grows and sustains relationships with various CBOs and rural networks, often crucial partners in recruiting for CE Studios. In our approach to CE Studios staffing, 1 or more of the 3 roles (facilitator, notetaker, or support person) is also a CEO navigator.

Since the CE Studios launch, 14 CE Studios for 11 different project teams have been conducted (Table 3). Over the past 3 years, 112 community experts participated in a CE Studio. One notable CE Studio took place on research examining safe firearm storage. Seven firearm owners of various ages, genders, and backgrounds (eg, law enforcement, hunter education, self-defense, gun clubs, 4-H) across Maine gathered to review and provide feedback on a survey being disseminated to firearm owners across NNE. Feedback from the CE Studios impacted language, definitions, and survey scope. Another CE Studio supported a clinical trial focusing on a social psychological intervention for patients with diabetes and occurred during grant preparation and before protocol finalization. The timing of this CE Studio allowed recommendations to influence many decision points, including the project’s name and overall language, eligibility criteria, and timeline and location of the intervention. In this instance, CE Studio participants were then invited to become part of the project’s longer-term community advisory board.

### 3.1. CE studios evaluation

As part of the CE Studios program evaluation at MHIR, community experts and researchers were asked to complete a follow-up survey about their experience. These surveys were adapted from the original team at Vanderbilt.^4^ At the time of writing this manuscript, 5 researchers and 22 community experts completed their respective surveys.

**Fig. 2.**
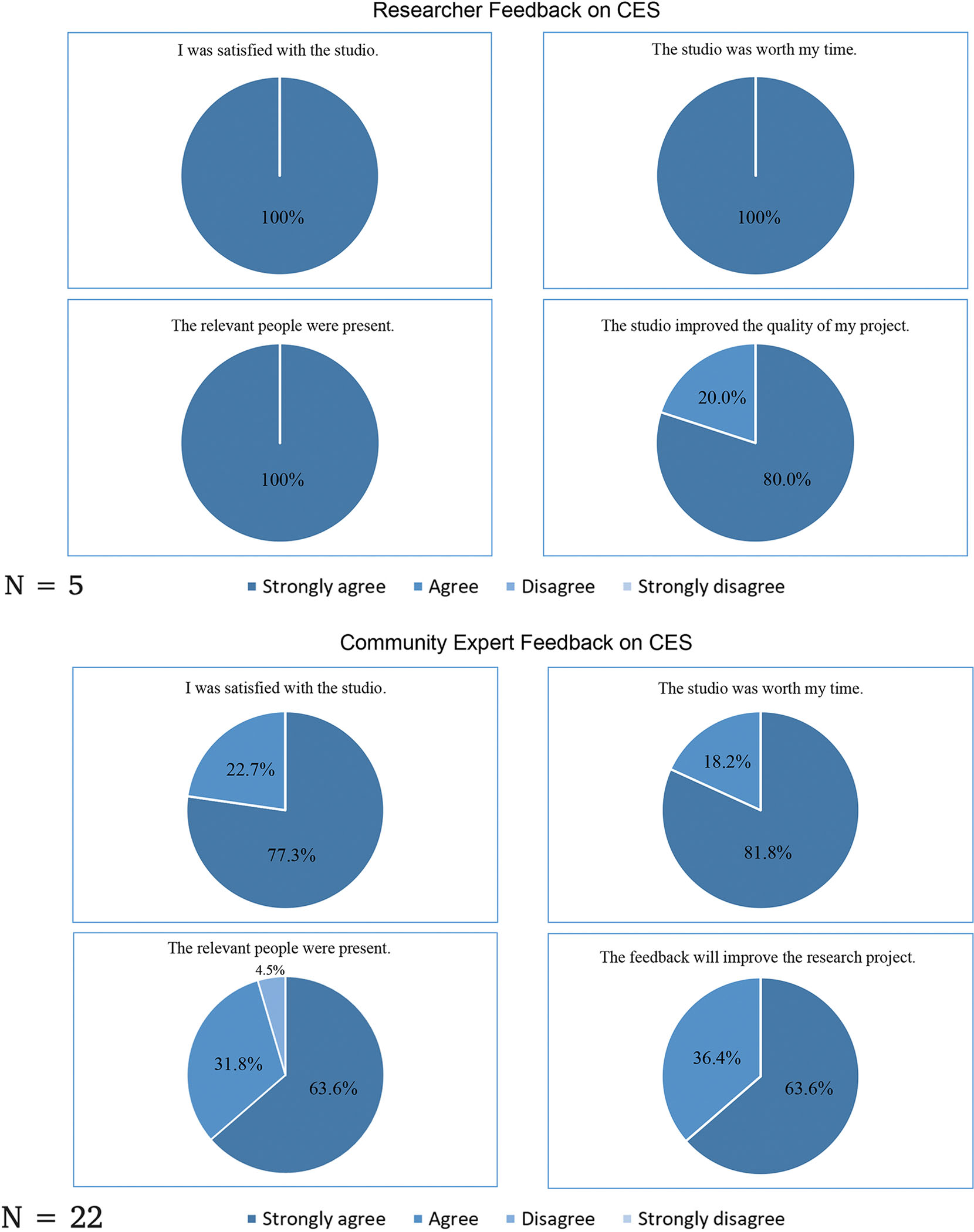
Researcher (A) and Community Expert (B) Follow-Up feedback after participating in a CE Studio, October 2022 to June 2025. Data represents community experts feedback from 6 of 14 completed CE Studios. Eight studios were not included in the evaluation because they were (1) completed in collaboration with a CE Studios team at Tufts University and feedback was collected through Tufts University feedback form (2 CE Studios); (2) in progress at the time of this paper and not included (4 CE Studios); or (3) adapted versions of the CE Studios model and excluded from the evaluation (2 CE Studios). CE, Community Engagement.

All researchers “strongly agreed” they were satisfied with the CE Studios, believed it was worth their time, and that the relevant community experts were present. Community experts also reported satisfaction with the CE Studios and that their time was well spent, although a notable portion “agreed” rather than “strongly agreed.” Most community experts agreed that the right people were in the room, except for 1 respondent who disagreed on this item. Feedback on whether the CE Studios would improve the research project was positive across both groups; however, “strong agreement” was less than prior items (Fig. 2).

### 3.2. CE studios lessons learned

As the CE Studios program at MHIR has grown, several lessons have emerged (Table 4). One lesson is the need to provide the research team with early clarity on (1) what a CE Studio is and is not, (2) when it is appropriate to apply, (3) and what are the roles and responsibilities. Without this clarity, misunderstandings on expectations (eg, timeline, publish-ability, amount of engagement) and misapplication (eg, use in place of a focus group or community advisory board) are apt to occur. The CE Studios team has also found that the CE Studios method can be overly rigid and, when used outside of broader CER frameworks, do not necessarily grow longer-term partnerships. The strong interest from MHIR research teams to date has highlighted the need to clarify (1) how this approach fits within a broader set of community engagement strategies, (2) what sustainable funding and staffing look like, and (3) how CE Studios can be most effectively integrated into the larger ecosystem of CER services and resources. Although NNE-CTR funds have supported implementation and some of the initial CE Studios, investigators are being encouraged to write CE Studios into grants to ensure funding and capacity for the work going forward.

Many logistical and administrative lessons have been learned during implementation. For example, during 1 CE Studio, a much higher number of participants than the team anticipated from the interest forms joined the activity. Although they were enthusiastic to have double the expected number of voices, this higher number led to challenges in ensuring participants received payment, providing technology support without contact information, and managing facilitation. Now the CE Studios team only shares the Zoom link just before the event, requests that participants not forward or share the link, ensures contact with all participants before the activity, and has extra gift cards available.

Another challenge is that recruitment timelines were extremely variable, with some CE Studios taking a few weeks and others months. With certain communities (eg, youth, new Mainer’s, firearm owners), having an in-person recruitment and/or warm hand-off from a CBO/trusted partner was crucial. In other instances, using strategic social media or other platform posts was an effective recruitment strategy (although necessary to screen for bots). To address this challenge, early communication about expectations with research teams started to occur, and the CE Studios team got more creative, reflexive, and responsive during recruitment. Retention and technology setup was also occasionally a challenge. With guidance from other regional institutions, the team adopted the compensation amount of $50 per hour, which seemed to help with recruitment and retention. Other strategies the team developed were regular and early communication with participants and highly personalized, flexible, and responsive tech support. In 1 CE Studio involving community members who had experienced financial distress due to cancer care, many participants were older adults living rurally in particularly vulnerable situations. To support participation, a staff member arranged to meet with participants to help them download and set up Zoom on their respective devices.

### 3.3. Limitations

Although there has been positive feedback on CE Studios from both researchers and community experts, we acknowledge that there were several limitations. For example, we had a relatively low response rate from community experts and a small pool of investigators eligible to complete the feedback survey. Another limitation was a lack of a 1-year or 2-year follow-up evaluation. Without these data, we are missing an understanding of lasting impacts of the CE Studios on projects and whether this activity builds capacity for future research engagement among community experts and community engagement among research partners.

### 3.4. Conclusions and future directions

Since their introduction at MHIR, CE Studios have been favorably experienced by both researchers and community experts. There has been growing interest in CE Studios by investigators and project leads across the institution and the region, particularly as a tool to engage rural voices. The high demand has shed light on the need to further develop the CE Studios infrastructure as it relates to staff time, training, mechanisms for community expert payment, and equitable access to the service across MHIR.

As part of growing this program and folding this work into a larger CER infrastructure, the CE Studios team at MHIR is exploring how CE Studios can be adapted to build capacity for future CER among researchers and community experts. They are also putting together a longer-term follow-up evaluation and outreach. Furthermore, they are working to clearly delineate community engagement mechanisms across the engagement continuum so that research teams can more easily select methods aligned with their project goals and scope.

## Figures and Tables

**Table 1.** Roles during community engagement studios.

Role	Responsibilities

Research team	• Presents the research in an understandable way for community experts • Answers clarifying questions for community experts
Facilitator	• Moderates and creates a welcoming environment • Guides the discussion based on the facilitation guide • Remains neutral • Encourages everyone to participate in the discussion
Notetaker	• (In-person) Takes notes on a flip chart or whiteboard • (Virtual) Takes notes on a document • Does not take verbatim notes on the community expert discussion, but takes brief (detailed) notes
Support	• (Virtual) Manages technology (Zoom, Microsoft Teams, and/or Google Meet) • Orients community experts to technology, as needed • Assists in any other technology issue
Community experts	• Digests the research presentation and asks the research team questions for clarification • Actively participates in the discussion to provide feedback on the research project • Draws recommendations from their lived experience, geographic location, and/or understanding of the community of interest

**Table 2.** Distinguishing community engagement studios from focus groups and research studios.

Component	Community Engagement Studio	Focus group	Research studio

Discussions	Bidirectional discussions	Unidirectional discussions	Bidirectional discussions
Audience	Community experts	General public audience	Research community and methodological experts
Purpose	Inform development, implementation, or dissemination *of* research	Use methods to collect qualitative data for research	Inform study design, implementation plans, and analytic approaches of research
Recruitment	Recruitment can come from surveys, community networking and engagement, and/or a repository of people who are interested	Recruitment can come from surveys, community networking and engagement, and/or a repository of people who are interested	Recruitment can come from existing research networks
Informed consent	Community experts serve as consultants and do not undergo informed consent	Participants must undergo informed consent	Research experts serve as consultants and do not undergo informed consent
IRB approval	Does not require IRB approval	Requires IRB approval	Does not require IRB approval
Dissemination	Discussions are summarized from notes and approved by community experts	Discussions can be recorded, transcribed, and analyzed	Discussions are summarized but may not be disseminated to participants
Recommendations	Recommendations are created for research design and development	Analysis of focus groups are compiled for research papers/outcomes	Recommendations are created to improve scientific rigor and feasibility of the research

Abbreviation: IRB, institutional review board.

**Table 3.** Community engagement studios facilitated at MHIR, October 2022 to June 2025.

Project name	CE Studios, No.	Lived experience of community expert	Area of CE Studios recruitment*	Community experts consulted, No.

Cost transparency in cancer care	1	Financial distress due to cancer care in NNE	Maine	10
Mitigating stigma in diabetes care	1	Having type II diabetes and receiving care in Maine	Maine	8
Research evidence to action	2	Making policy decisions guided by health research	Maine	8
Sports equity	1	Mentoring, coaching, parenting, or direct experience of New Mainer high school girls’ engagement in sport or physical activity	Lewiston and Portland, Maine	18
Safe firearm storage	1	Owning a firearm and living in NNE	NNE	7
Dual vaccine	1	Rural individuals eligible for dual vaccine	NNE and Tufts University	7
Infant and baby monitoring	1	New parents in NNE	NNE	7
Feeding families	1	Raising children and navigating food insecurity in Maine	Maine	10
MERGE: improving rural obstetrics care	3	Receiving rural obstetric care in Maine or New Hampshire	Maine and New Hampshire	26
HOPE dissemination	1	Working in positions relevant to HOPE dissemination	NNE and Tufts University	8
Mindful rest	1	Caretakers of youth with autism who struggle with sleep	NNE	4
Total	14			112

Abbreviations: CE, Community Engagement; HOPE, Healthy Outcomes from Positive Experiences; MERGE, Maine Rural Graduate Medical Education Collaborative; NNE, Northern New England.

* All occurred virtually.

**Table 4.** Lessons learned from implementing community engagement studio infrastructure at MHIR.

Themes	Topics	Lessons learned and recommendations

Training	Community of practice	Establish a community of practice and mentorship relationships where possible. Build on what has already been done at other institutions.^8^
	Roles	Ensure that all processes and team roles are well-understood, responsibilities are accounted for, and channels of communication are clear.
	Facilitation	New team members can benefit from participating in at least 2 CE Studios in supporting roles (eg, notetaker, support) before taking on a facilitator role. A background in facilitation or additional facilitation training is also recommended.
	Training and resources	Provide training and develop a toolbox of resources, documents, and example forms when implementing CE Studios.
Building capacity and sustaining relationships	Building researcher capacity	Researcher participation in CE Studio implementation can support researchers in learning the value and applicability of CE Studios and CER.
	Meaningful engagement	Ensure CE Studios are focused on topics that can change based on community expert feedback.
	Continual engagement	Consider creating appropriate pathways to share back and re-engage with community experts. Build capacity for future engagement.
	Build and earn trust	Ensure engagement with CE Studios is positive for lived-experience experts. Earn and build trust with community partners.
Sustaining CE Studios (financially)	Researcher funds	Have researchers include funds for community-engaged research activities, including CE Studios, in their grant budgets.
	Institutional support	Protect time for community-engaged research personnel. Track time, personnel, and income to share data about staffing needs with leadership.
Setting expectations and clarification on method	Appropriate use of CE Studios	Ensure each research team understands the purpose of the CE Studios, the reason for the format and structure, and appropriate dissemination and application of CE Studio recommendations.
	Contextualizing the CE Studios method within the larger CER framework	Provide education and clear communication about the consultative nature of CE Studios and where they fit on the spectrum of engagement.
	Clarify method	Build tools and resources to help clarify methods and approaches for researchers (eg, CE Studios, research studios, focus groups).
	Set expectations	Make and set realistic expectations about the capacity and timeline of CE Studios and what is achievable in a single CE Studio. Refrain from an excessive number of CE Studios.
Administrative tasks	Staff capacity	Have early and realistic discussions with research and department administrative team about CE Studios staffing, time, funding, and sustainability needs.
	Zoom technology	Consider what remote platform will be used and the familiarity and accessibility of that platform to the research team and community experts. Ensure recording, permissions, and waiting rooms are all set up appropriately before the CE Studios. Send the Zoom link just before the meeting and remind participants not to share the link.
	Compensating community experts	Ensure that community experts are compensated for their time. Plan well in advance to ensure the mechanisms and pathways for payment are established. (It can be useful to have extra gift cards. The MHIR team used a compensation rate of $50 per hour, matching other regional institutions’ rate for community expert activities.)
Reducing power dynamics between the researcher/team and participants	Setting the stage	Ensure lived-experience expertise is valued throughout the CE Studios. Emphasize that participants are welcome to share any thoughts or feedback without judgment from the team.
	Facilitator and researcher(s) should be neutral	Provide training resources with tips on how to use neutral language during CE Studios. Prepare researchers for the expectations of how they will interact with community experts.
	Bidirectional learning	Share with participants that the researchers and team are there to learn from community experts, and that community experts may learn from researchers and team, too.
Recruitment and retention strategies	Use multiple methods of recruitment	Consider and be responsive to the unique recruitment needs for each CE Studio. Some approaches may include using social media platforms, Craigslist, flyers, word-of-mouth from community members, researching and cold-calling relevant interest holders, and leaning into partnerships with CBOs, schools, and other networks to recruit participants. Some recruitment may involve going in-person to establish trust.
	Set recruitment expectations	Plan for a longer recruitment period when recruitment in rural areas or among certain populations may be slow.
	Participant retention	Establish personal contact with participants before the CE Studios. Send a reminder in the week before and on the day of the CE Studio. Allow the technology support person to call or text “no shows.”

Abbreviations: CBO, community-based organization; CE, Community Engagement; CER, community-engaged research.
